# Immune checkpoint inhibitors and type 1 diabetes mellitus: a case report and systematic review

**DOI:** 10.1530/EJE-19-0291

**Published:** 2019-07-19

**Authors:** Jeroen M K de Filette, Joeri J Pen, Lore Decoster, Thomas Vissers, Bert Bravenboer, Bart J Van der Auwera, Frans K Gorus, Bart O Roep, Sandrine Aspeslagh, Bart Neyns, Brigitte Velkeniers, Aan V Kharagjitsingh

**Affiliations:** 1 Department of Endocrinology, Universitair Ziekenhuis Brussel, Brussels, Belgium; 2 Diabetes Clinic, Universitair Ziekenhuis Brussel, Brussels, Belgium; 3 Department of Medical Oncology, Universitair Ziekenhuis Brussel, Brussels, Belgium; 4 Medical Library, Haaglanden Medical Center, Hague, The Netherlands; 5 Diabetes Research Center, Vrije Universiteit Brussel, Brussels, Belgium; 6 Department of Immunohematology & Blood Transfusion, Leiden University Medical Center, Leiden, The Netherlands; 7 Department of Diabetes Immunology, Diabetes & Metabolism Research Institute, City of Hope, Duarte, California, USA; 8 Section Endocrinology, Department of Internal Medicine, Leiden University Medical Center, Leiden, The Netherlands

## Abstract

**Objective:**

To better define the rare adverse event (AE) of diabetes mellitus associated with immune checkpoint inhibitors (ICIs).

**Design and methods:**

We report the case of a lung cancer patient with diabetic ketoacidosis (DKA) and autoimmune thyroiditis during pembrolizumab treatment. We provide a systematic review of all published cases (PubMed/Web of Science/Cochrane, through November 2018) of autoimmune diabetes mellitus related to blockade of the cytotoxic T-lymphocyte antigen 4 (CTLA-4)-, programmed cell death 1 (PD-1) receptor or its ligand (PD-L1) or combination (ICI) therapy.

**Results:**

Our literature search identified 90 patient cases (our case excluded). Most patients were treated with anti-PD-1 or anti-PD-L1 as monotherapy (79%) or in combination with CTLA-4 blockade (15%). On average, diabetes mellitus was diagnosed after 4.5 cycles; earlier for combination ICI at 2.7 cycles. Early-onset diabetes mellitus (after one or two cycles) was observed during all treatment regimens. Diabetic ketoacidosis was present in 71%, while elevated lipase levels were detected in 52% (13/25). Islet autoantibodies were positive in 53% of patients with a predominance of glutamic acid decarboxylase antibodies. Susceptible HLA genotypes were present in 65% (mostly DR4). Thyroid dysfunction was the most frequent other endocrine AE at 24% incidence in this patient population.

**Conclusion:**

ICI-related diabetes mellitus is a rare but often life-threatening metabolic urgency of which health-care professionals and patients should be aware. Close monitoring of blood glucose and prompt endocrine investigation in case of hyperglycemia is advisable. Predisposing factors such as HLA genotype might explain why some individuals are at risk.

## Introduction

Unleashing the power of the immune system with monoclonal antibodies targeting immune checkpoint receptors has been a major breakthrough causing a paradigm shift in the treatment of many types of cancer. The deficient anti-tumor immune response can be restored by blocking inhibitory immune receptors of which cytotoxic T-lymphocyte antigen 4 (CTLA-4), programmed cell death 1 receptor (PD-1) and its ligand (PD-L1) have become part of our standard of care options in many indications (1). Immune checkpoint blockade is associated with a unique risk for immune-related AEs (irAE), affecting the endocrine organs in 4–30% of patients (2, 3). While hypophysitis and thyroid disorders are the most frequent endocrine irAE, autoimmune diabetes mellitus is a rare (1%) but potentially life-threatening irAE deserving further notice (4). It appears more frequently with PD-1 or PD-L1 blockade (or combination therapy) than with anti-CTLA-4 (ipilimumab) therapy (5, 6), highlighting the importance of the PD-1/PD-L pathway in maintaining self-tolerance against pancreatic islets. Similarities with ‘classic’ type 1 diabetes mellitus (T1D) include the presence of islet antibodies and susceptible HLA genotypes (4, 6). The clinical significance of diabetes mellitus associated with checkpoint blockade is estimated to increase as these novel anticancer agents are both initiated to a greater extent and at an earlier disease stage (7). We describe a patient with rapid-onset diabetes mellitus and ketoacidosis associated with the ICI pembrolizumab (anti-PD-1). We subsequently performed a systematic review and present an overview of similar cases of diabetes mellitus related to CTLA-4, PD-1, PD-L1 or a combination of CTLA-4 and PD-1 checkpoint inhibitors. We discuss the clinical presentation, potential mechanisms and suggestions for optimal management.

## Case report

Our patient is a 61-year-old male with a recent diagnosis of metastatic non-small-cell lung carcinoma (NSCLC). Eight weeks after initiating treatment with pembrolizumab, he presented at the emergency department with a 5-day history of nausea, vomiting, diarrhea and generalized weakness. He had no personal or family history of endocrine or autoimmune disease. Physical examination revealed impaired consciousness, dry mouth, marbled skin and cold extremities. He was hypotensive (105/45 mmHg) and tachycardic (108/min). Blood analysis showed a marked hyperglycemia (1194 mg/dL = 66.3 mmol/L), pseudohyponatremia (117 mmol/L – corrected 143 mmol/L) (8) and acute renal insufficiency (CrCl 28 mL/min/1.73 m^2^). The positive reaction for urinary ketones and a blood gas analysis showing severe metabolic acidosis with respiratory compensation, established the diagnosis of diabetic ketoacidosis. The patient was hospitalized at our intensive care unit for monitoring, rehydration and intravenous insulin therapy. He recovered and was switched to a subcutaneous basal-prandial insulin regimen. An autoimmune etiology was probable, given the context and the presence of positive glutamic acid decarboxylase autoantibodies (GADAs) with low C-peptide levels (Table 1). The serum lipase level was also elevated at diagnosis (>3 times the upper reference limit). Abdominal computed tomography did not show signs of pancreatitis. The HLA class II genotype of our case was assessed by allele-specific oligonucleotide hybridization, as previously described (9). HLA genotype analysis identified homozygosity for the haplotype DRB1*04-DQA1*03:01-DQB1*03:02 (DR4-DQ8). Subclinical hyperthyroidism was simultaneously detected (TSH 0.058 mIU/L, fT4 18.7 pmol/L) which evolved into manifest hypothyroidism (TSH 18.92 mIU/L, fT4 5.7 pmol/L) over the next weeks requiring levothyroxine substitution therapy. Ultrasonography of the thyroid did not demonstrate hypervascularity, and thyroid autoantibodies (TPOAb, TSI) were negative. This clinical pattern was suggestive of checkpoint blockade-induced thyroiditis (10, 11, 12, 13). We subsequently performed a systematic review to identify similar cases of diabetes mellitus associated with ICI.

**Table 1:** Laboratory data on admission.

	Value	Ref.
Blood		
Glucose, mg/dL	1194	70–100
Urea, mg/dL	96	19–43
Creatinine, mg/dL	2.4	0.66–1.25
eGFR (MDRD; mL/min/1.73 m^2^)	28	>60
Na, mmol/L	117	137–145
K, mmol/L	5.6	3.4–5.0
Cl, mmol/L	86	98–107
HCO_3_, mmol/L	6	22–30
Anion Gap, mmol/L	31	10–18
Ca, mmol/L	1.86	2.10–2.50
P, mmol/L	0.77	0.81–1.45
Mg, mmol/L	0.95	0.66–0.95
Alb, g/L	31	35–50
CRP, mg/L	100.3	<5
LDH, U/L	408	313–618
AST, U/L	16	17–59
ALT, U/L	29	21–72
ALP, U/L	113	38–126
GGT, U/L	36	<73
Bili, mg/dL	0.43	0.2–1.3
Lipase, U/L	970	23–300
Platelet, ×10^3^/mm^3^	305	158–450
Hb, g/dL	14.3	13.0–16.5
WBC, ×10^3^/mm^3^	21.9	3.6–9.6
Neutrophils, %	86.0	41.0–74.0
(Absolute), ×10^3^/mm^3^	18.834	1.4–6.7
Urine		
Protein	Negative	
Glucose, g/L	++++	
	44.3	
Creatinine, mg/dL	25	
Na, mmol/L	10	
Osm, mosmol/kg H_2_O	418	
Arterial blood gas		
pH	6.944	7.35–7.45
PaCO_2_, mmHg	18.5	36–44
PaO_2_, mmHg	86.5	65–80
HCO_3_, mmol/L	4.0	22–26
Base excess, mmol/L	−26.2	−2, +2
Lactate, mmol/L	1.6	0.7–2.1
Thyroid (before the start of immunotherapy)		
TSH, mIU/L	1.11	0.27–4.2
fT4, pmol/L	12.4	11.0–24.0
Diabetes		
ICA	Negative	
Insulin Ab, % binding	0.5	<0.6
GADA, WHO U/mL	>171	<23
IA2A, WHO U/mL	<0.1	<1.4
C-peptide, nmol/L	0.02	0.29–0.99

## Methods

Several databases (PubMed/Web of Science/Cochrane) were searched through November 2018, for case reports on the subject of diabetes mellitus and checkpoint inhibitors, by two reviewers independently (J M K d F and A V K). The investigators screened the title and abstract for manuscript selection. Language was restricted to English. Congress reports were excluded. Supplementary Table 1 (see section on supplementary data given at the end of this article) provides an overview of our search terms. Additionally, the authors reviewed the reference lists of the included articles (4, 13, 14, 15, 16, 17, 18, 19, 20, 21, 22, 23, 24, 25, 26, 27, 28, 29, 30, 31, 32, 33, 34, 35, 36, 37, 38, 39, 40, 41, 42, 43, 44, 45, 46, 47, 48, 49, 50, 51, 52, 53, 54, 55, 56, 57, 58, 59, 60, 61, 62, 63, 64, 65, 66, 67, 68) and identified five additional cases (69, 70, 71, 72). The following data were extracted from each manuscript: author, year of publication, age, gender and ethnicity of the patient, cancer type, checkpoint inhibitor therapy, number of cycles of therapy, prior immunotherapy, relevant past medical history (PMH), presence of diabetic ketoacidosis, glycemia, glycated hemoglobin, C-peptide, islet autoantibodies, lipase, other irAE and HLA genotype. The number of treatment cycles was preferred rather than the time to onset in weeks, as this information was not consistently available (immune checkpoint therapy is usually given every 3 weeks). We categorized the HLA haplotype into three classes: ‘susceptible’, ‘neutral’ or ‘protective’ for autoimmune diabetes. Specifically, haplotypes were categorized as susceptible in the presence of (1) HLA A2, DR3, DR4 or (2) the presence of DR9 in a Japanese population or (3) when the authors of the original paper had categorized it susceptible. They were protective in case of DQ6, DR11 or DR16-DQ5. Written informed consent for genetic analysis and publication was obtained from our patient.

## Results

**Figure 1:**
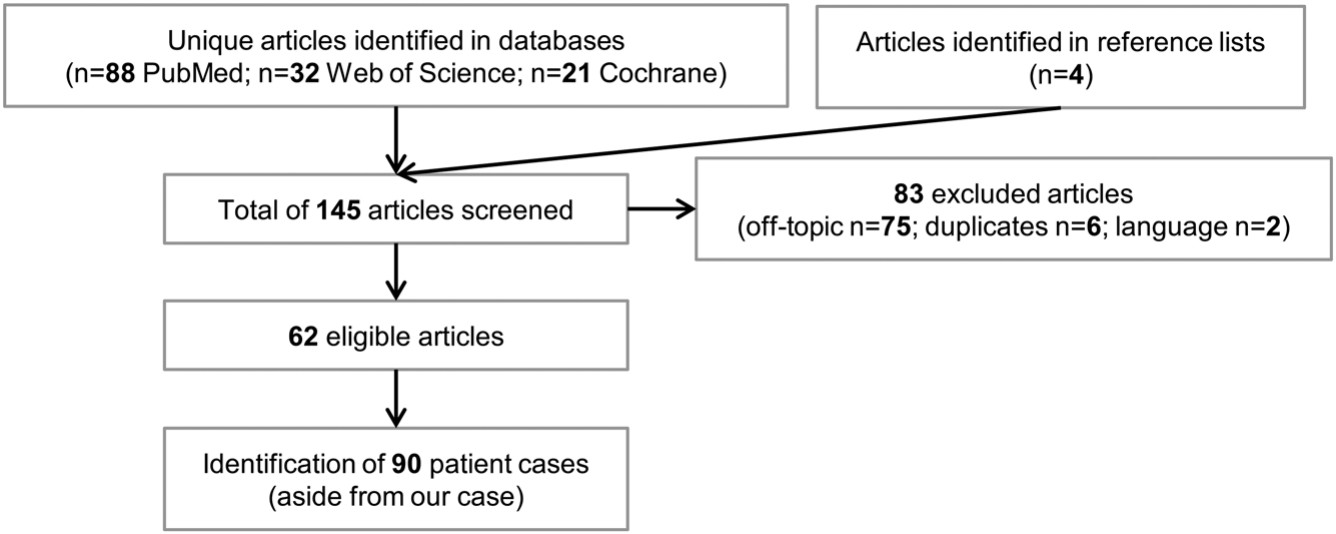
Flow chart of study selection.

Our search identified a total of 145 articles of which 62 were eligible. Figure 1 shows a flow chart of the study selection. We identified a total of 90 cases, aside from our patient, with a male predominance (55/91, 60%) and a mean age of 61 years (range 22–84). The ethnicity was Asian in 15%. The main tumor types were melanoma (48/91, 53%) and NSCLC (14/91, 15%). Relevant PMH, namely diabetes mellitus, thyroid disease or risk thereof, was noted in 22% (20/91). One in four cases (22/91, 24%) had received prior immunotherapy, with IL-2 (2/91), interferon (7/91), ipilimumab (16/91) and/or nivolumab (3/91). The different treatment regimens included monotherapy with anti-CTLA-4 (3/91, 3%), anti-PD-1 (65/91, 71%), anti-PD-L1 (7/91, 8%) or a combination of anti-CTLA-4 with anti-PD-1 (14/91, 15%). One patient received PD-L1 with 4-1BB (CD137) blockade and one other patient received either CTLA-4 or PD-1 blockade therapy. Thus, the treatment regimen mostly observed in this cohort was anti-PD-1 monotherapy; blockade of the PD-1/PD-L pathway was involved in 96% (87/91). Only three cases of diabetes development were observed during anti-CTLA-4 monotherapy. Importantly, all of these were pre-treated with nivolumab (2/3) and/or interferon (2/3). On average, patients were diagnosed with diabetes after 4.5 treatment cycles (range: 1–17), while this appeared to be earlier for the combination of anti-CTLA-4 and PD-1 therapy (2.7 cycles, range: 1–5). Cases of early-onset diabetes (after only one or two cycles) were observed in all treatment regimens. The presentation of diabetes mellitus related to checkpoint blockers often follows a severe course. Seventy-one percent of patients (64/91) presented with diabetic ketoacidosis (DKA), with a median presenting glycemia of 565 mg/dL (range: 209–1211) and glycated hemoglobin of 7.6% (average: 7.7%; range: 5.4–11.4). Low C-peptide levels were present at diagnosis in 84% (58/69) of cases. The onset appeared earlier for patients presenting with DKA, with 4 versus 5.9 cycles. Elevated lipase levels were detected in 52% (13/25) of analyzed patients. At least one of the islet autoantibodies was positive in 53% (47/88), while two or more autoantibodies were detected in 15% (13/88). The autoantibody analysis was positive in 51% of patients for GADA, 18% for insulinoma-associated antigen-2 (IA-2), 13% for islet-cell antibodies (ICA), 26% for anti-insulin and 4% for zinc transporter 8 (ZnT8). Table 2 shows an overview of pancreatic autoantibodies. The mean time of onset was 3.1 cycles (range: 1–17) for GADA-positive and 5.9 cycles (range: 1–16) for GADA-negative patients. The genetic HLA region was analyzed in 56% (51/91) of patients. Genotypes susceptible for T1D or fulminant diabetes were present in 61% (31/51), while a protective genotype was simultaneously present in an additional 4% (2/51). The DR4, DR3, DR9 and A2 were the dominant HLA serotypes. Table 3 shows an overview of the HLA genotypes. Thyroid dysfunction related to checkpoint inhibition (thyroiditis, primary hypo- or hyper-thyroidism) developed in 24% (21/91) of patients. Of these 21 patients, two had a known history of hypothyroidism. A summary of the results can be found in Table 4.

**Table 2:** Pancreatic autoantibodies and ICI-induced diabetes.

	All	GAD	ICA	IA-2	Insulin	ZnT8
Present	47	43	3	10	9	1
Absent	41	42	20	45	26	23
N/A	3	6	68	36	56	67
Frequency (%)	53	51	13	18	26	4

GAD, glutamic acid decarboxylase; IA-2, insulinoma-associated antigen-2; ICA, islet-cell antibodies; N/A, not available; ZnT8, zinc transporter 8.

**Table 3:** HLA genotype in patients with ICI-induced diabetes.

HLA genotype		Serotype	#
Susceptible	31/51 (61%)	A2.1	5
Susceptible and protective	2/51 (4%)	DR3	8
Neutral	10/51 (20%)	DR4	23
Protective	8/51 (16%)	DR9	5
N/A	40/91	DR4-DQ4	2
		Other	15

ICI, immune checkpoint inhibitor; N/A, not available.

## Discussion

We present a comprehensive overview of diabetes mellitus development in patients treated with ICIs and describe a patient with simultaneous rapid onset of diabetes mellitus with ketoacidosis and thyroiditis associated with the checkpoint inhibitor pembrolizumab. We confirm that diabetes mellitus is an important, yet rare, side effect. Similar to our case, these patients often present with a fulminant onset of diabetes mellitus and the presence of ketoacidosis at the time of diagnosis (4, 6, 15). Its onset ranges from a few weeks, sometimes even after the first or second cycle of immunotherapy (4, 6), up to more than a year after the initiation of immunotherapy (6, 17). We observed the early pattern of diabetes onset with all classes of checkpoint inhibitors. The onset of β cell inflammation is often fulminant, suggested by the relatively low glycated hemoglobin levels, while C-peptide levels are usually low or undetectable at diagnosis. This irAE is predominantly found in patients exposed to blockade of the PD-1/PD-L pathway. We further observed that a quarter of all patients has received prior immunotherapy, and it is possible that this influenced the results, as it was previously observed that the combination of rapamycin and IL-2 transiently decreased C-peptide levels in T1D patients (73), and additionally T1D was also a reported side effect of interferon therapy (74). Islet autoantibodies were detected in half of patients, with GADA being the predominant antibody, although it should be noted that the other autoantibodies were not as systematically analyzed. This differs from ‘classic’ T1D where autoantibodies are present in 80–95% of patients (75, 76). It has previously been suggested that the presence of autoantibodies at the time of diagnosis is related to an earlier onset of ICI-induced diabetes (4, 6, 43, 58). Our review supports this hypothesis. It would be of academic value to prospectively investigate this phenomenon as well as the serologic status of non-diabetes patients in future studies. Additionally, there was biochemical evidence of pancreatic inflammation with elevated lipase levels reported in about 50% of all cases. Several authors also described radiographic changes in the pancreatic volume during immunotherapy, notably pancreatic enlargement before diabetes onset, followed by a volume decrease (14, 46). These findings are often asymptomatic (6, 14) as was the case in our patient. It remains unclear to which extent the pancreatic exocrine gland is involved. It has been hypothesized that ‘classic’ T1D is in fact a combined endocrine–exocrine disease in which the loss of functional β cell mass is most clinically apparent (77) and non-specific elevations of amylase and lipase occur in 16–25% of cases with DKA (78). In the context of checkpoint blockade therapy, asymptomatic elevations of lipase and/or amylase have also been reported in the absence of new-onset diabetes (79, 80). Whether these patients are prone to develop diabetes in the future is an additional question to address in prospective studies.

The strength of our study is the broad and extensive investigation with the exploration of different search engines. Our study also has its limitations. The analysis included individual patient data, of whom not all parameters of interest were available. The incidence could not be calculated by the lack of the total number of treated patients. We do believe however that this AE is still underestimated as it is increasingly being reported, as shown by a recent pharmacovigilance study (81). The incidence of DKA was lower (50.2 vs 71%) in their analysis. Another research group described a large case series of 27 patients with insulin-dependent diabetes induced by checkpoint inhibitors. Compared to our analysis, they also reported DKA less frequently (59 vs 71%) (6). This might be due to a publication bias in our study toward fulminant presentations, due to an underrepresentation of milder diabetes cases in the literature.

**Figure 2:**
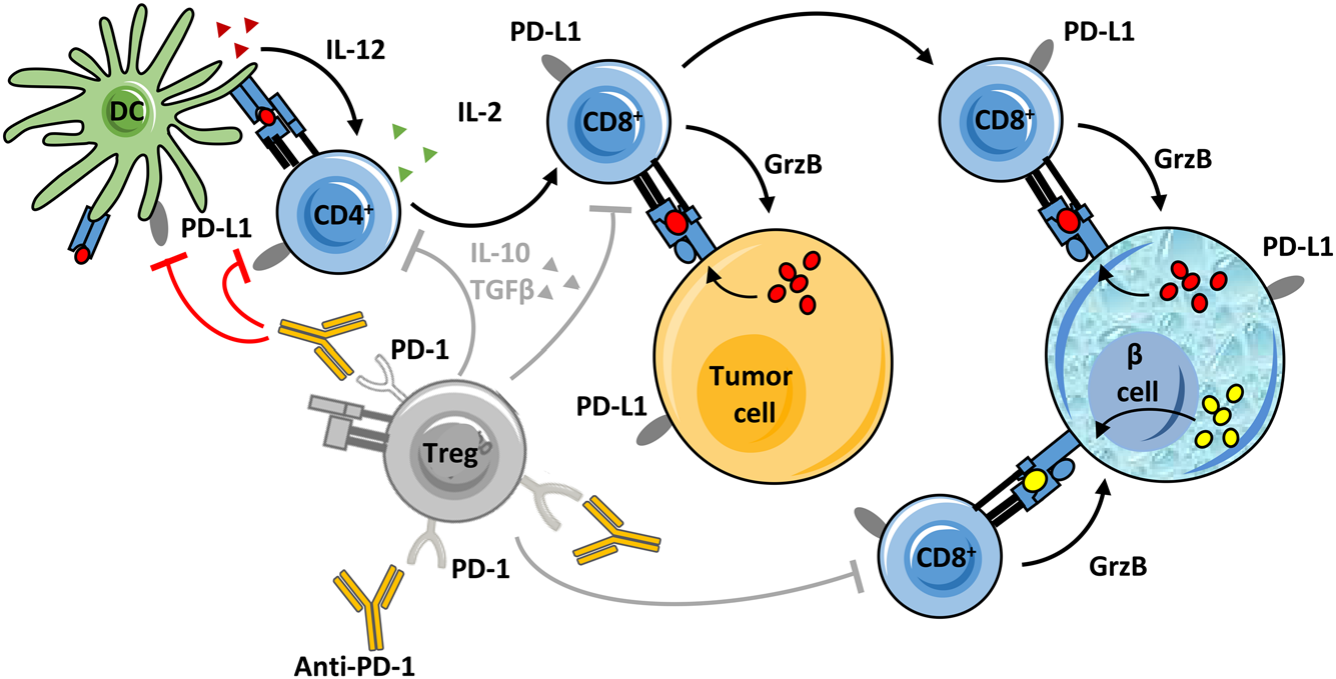
Pathophysiology of immune checkpoint inhibitor-associated diabetes mellitus.

The role of immune checkpoints in the pathophysiology of diabetes mellitus has been investigated in mice and in humans. Non-obese diabetic (NOD) mice develop rapid-onset diabetes following the blockade of PD-1 or PD-L1 but not PD-L2 (82). This corresponds with the finding that pancreatic islets express PD-L1 at low levels in mice (dramatically upregulated in inflamed islets), while PD-L2 expression is not detected (82, 83). Definite conclusions remain difficult as PD-L1 also binds to B7-1 (CD-80), itself a ligand for CD-28 and CTLA-4 (84). Specific blockade of the PD-L1:B7-1 interaction preferably induced diabetes in older (13 weeks old) as compared to younger (6–7 weeks old) NOD mice, while the blockade of both PD-L1: PD-1 and PD-L1:B7-1 interactions rapidly induced diabetes in mice of both ages (85). This suggests a multi-faceted role for PD-L1 in diabetogenesis. To the best of our knowledge, there is no evidence of CTLA-4 expression on pancreatic islets, although the transgenic overexpression of anti-CTLA-4 Fv on β cells could protect NOD mice from autoimmune diabetes (86). In humans, polymorphisms in the CTLA4 and PD-1 gene confer increased susceptibility to a variety of autoimmune disorders, including T1D (87, 88, 89, 90, 91, 92). The CTLA-4 and PD-1/PD-L pathways have been studied in T-cell subsets from patients with ‘classic’ T1D. Both decreased PD-1 gene expression in peripheral CD4^+^ T cells (93) as a low frequency of circulating PD-1^+^ CD4^+^ T cells were found in T1D patients (94). More recently, Granados *et al*. demonstrated further PD-1 dysregulation as activated peripheral T cells from children with new-onset T1D failed to upregulate PD-1 upon T-cell receptor stimulation (95). Regulatory T cells (T_regs_) express both CTLA-4 (96) and PD-1 (97), essential in their activation and suppressive role in peripheral immune tolerance (97, 98, 99) and a deficiency in the ability to upregulate PD-1 and efficiently use the PD-1/PD-L pathway has been observed in CD4^+^ CD25^+^ T_regs_ from T1D patients (100). Furthermore, human pancreatic β cells express PD-L1, which is induced by IFN-γ (and to a lesser extent IFN-α). This expression is upregulated in inflamed islets and is associated with CD8^+^ T-cell infiltration (101, 102). One could hypothesize that β cells respond in such a way to attempt to suppress autoreactive CD8^+^ T cells. Figure 2 illustrates the pathophysiology of immune checkpoint inhibitor-associated diabetes mellitus.

Predisposing factors for ICI-induced diabetes should be better defined. ‘Classic’ T1D has a strong genetic component, with the HLA class II alleles accounting for up to 50% of the disease risk (103). Differences between populations and diabetic genotypes do exist, as the DR3-DQ2 and DR4-DQ8 haplotypes are a major risk factor for T1D (103), while the DR4-DQ4 and DR9-DQ9 haplotypes are linked with fulminant diabetes in Asians (104). Our patient was homozygous for the DR4-DQ8 haplotype, an HLA pattern associated with a high risk for type 1 diabetes. In this review, the majority of patients had a HLA genotype with increased susceptibility for either T1D or fulminant diabetes (61%), which is striking when compared to a Caucasian reference population (susceptible genotypes: 9.1%; rare, neutral or moderately protective/susceptible genotypes: 23.9%; protective genotypes: 67.1%) (105). There was also a predominance of HLA-DR4, similar to the cohort described by Stamatouli *et al*., although they reported a higher frequency (16/21, 76%) (6). We might consider checkpoint blockade-induced diabetes to be a distinct diabetes subtype, showing typical features of ‘classic’ T1D and fulminant diabetes. The rapid-onset of diabetes with ketoacidosis, relatively low glycated hemoglobin levels and pancreatic inflammation are suggestive of fulminant diabetes, while the seemingly non-Asian ethnical predominance and the presence of autoantibodies (although less frequent) fit to the ‘classic’ T1D. Alternatively, checkpoint blockade-induced diabetes mellitus may merely be a heterogeneous collection of variants of autoimmune diabetes, exhibiting the increasingly acknowledged heterogeneity of T1D (106). Table 5 compares the characteristics of the different diabetes subtypes (78, 107, 108, 109, 110, 111, 112, 113). Patients who received immune checkpoint therapy after pretreatment with other immunotherapy also appeared to be at increased risk. The combination of checkpoint blockade therapy carries an increased incidence of irAE as compared to monotherapy (114). In a meta-analysis by Barroso-Sousa *et al*., patients who received combination immunotherapy were more likely to develop thyroid dysfunction and hypophysitis (2). This may hold true as well for ICI-induced diabetes. Prior autoimmune disease, such as autoimmune thyroid disease (spontaneous or associated with immunotherapy) could be another potential risk factor for the development of diabetes mellitus related to ICI. The pre-existence of type 2 diabetes did not appear to be a particular risk factor in a French retrospective analysis (25). In this study, one patient with insulin-dependent type 2 diabetes had worsening glycemic control, although it could not be excluded that pancreatic autoimmunity already existed (i.e. LADA) before the start of nivolumab therapy (26).

**Table 4:** Summary of results.

Characteristic	All cases (*n* = 91)
Age, years	
Median (range)	61 (22–84)
Gender	
Female/male	36 vs 55
Ethnicity	
Asian	14/91 (15%)
Tumor types	
Melanoma	48/91 (53%)
NSCLC	14/91 (15%)
Past medical history*	20/91 (22%)
Prior immunotherapy	22/91 (24%)
IL-2	2/91
Interferon	7/91
Ipilimumab	16/91
Nivolumab	3/91
Immune checkpoint inhibitor	
Anti-CTLA-4	3/91 (3%)
Anti-PD-1	65/91 (71%)
Anti-PD-L1	7/91 (8%)
Anti-CTLA-4 + anti-PD-1	14/91 (15%)
Anti-PD-L1 + 4-1BB blockade	1/91
CTLA-4 or PD-1 blockade	1/91
Time-to-diagnosis in cycles (range)	4.5 (1–17)
Combination therapy	2.7 (1–5)
With/without DKA	4 vs 5.9
GADA pos./GADA neg.	3.1 vs 5.9
Diabetic ketoacidosis	64/91 (71%)
Glycemia, median (range)	565 mg/dL (209–1211)
Glycated hemoglobin, median (range)	7.6% (5.4–11.4)
Low-C-peptide at diagnosis	58/69 (84%)
Elevated lipase	13/25 (52%)
Positive pancreas autoantibodies	
At least one	47/88 (53%)
Two or more	13/88 (15%)
Type of pancreas autoantibodies	
GADA	51%
IA-2	18%
ICA	13%
Anti-insulin	26%
ZnT8	4%
HLA analysis	51/91 (56%)
Susceptible	31/51 (61%)
Susceptible and protective	2/51 (4%)
Neutral	10/51 (20%)
Protective	8/51 (16%)
Thyroid dysfunction with ICI	21/91 (24%)
Prior history of thyroid dysfunction	2/21

*Diabetes mellitus, thyroid disease or risk thereof.

4-1BB, CD137; CTLA-4, cytotoxic T lymphocyte antigen 4; DKA, diabetes ketoacidosis; GADA, glutamic acid decarboxylase; HLA, human leukocyte antigen; IA-2, insulinoma-associated antigen-2; ICA, islet-cell antibodies; ICI, immune checkpoint inhibitor; IL-2, Interleukin-2; NSCLC, non-small cell lung cancer; PD-1, programmed cell death protein 1; PD-L1, programmed death-ligand 1; ZnT8, zinc transporter 8.

**Table 5:** Classification of diabetes mellitus.

	Checkpoint blockade	‘Classic’ type 1	LADA	Fulminant diabetes
Clinical features				
Age at onset (range)	61 years (22–84)	Childhood or adolescence Rarely adult	>30 years	Adult
Ethnicity	Both	Non-Asian	Non-Asian	Asian
Symptoms at diagnosis	Acute (rarely subclinical)	Acute	Subclinical	Acute
Ketoacidosis	Yes (76%)	Possible	Rarely	Yes
Insulin required at diagnosis	Yes	Yes	No	Yes
Biochemical features (at diagnosis)				
C-peptide	Low or undetectable (84%)	Low or undetectable	Low or normal	Low or undetectable
HbA1c (range)	7.5% (5.4–11.4)	>6.35%	7.86	<8.7%
Lipase	Elevated (52%)	Elevated (24%, DKA)	?	Elevated (98%)
Autoantibodies	Positive (53%)	Positive (>80%)	Positive	Negative
Pathophysiology				
HLA association	Suspected	High risk	High/mild risk	High risk

References: (78, 107, 108, 109, 110, 111, 112, 113).

CRP, C-reactive peptide; HbA1c, glycated hemoglobin; LADA, latent autoimmune diabetes of adults.

Health care professionals should be aware of this possible side effect as these novel anticancer agents are increasingly used (7). Clinical signs and symptoms of hyperglycemia should be checked and when present, should prompt blood glucose measurement. Routine monitoring of blood glucose before each administration of ICI therapy is currently advisable (5, 115, 116). This would theoretically allow for an early diagnosis of glucose abnormalities and of DKA in particular. Some have even suggested to provide a glucometer to patients with a history of autoimmune disease (4). The usefulness of glucose monitoring has been disputed however in a retrospective analysis of fasting glycaemia in anti-PD-1-treated patients, suggesting that glucose monitoring does not allow to anticipate T1D in this patient population, perhaps due to its brisk onset (25). Upon the detection of new-onset diabetes or worsening glycaemia in patients with known type 2 diabetes mellitus, the glycated hemoglobin and pancreatic autoantibodies (especially GADA) should be analyzed to support the diagnosis of checkpoint blockade-related diabetes mellitus. Measurement of C-peptide is not strictly necessary for the diagnosis and treatment of this AE, especially given its fluctuation in time, although serially measured (or in the non-DKA phase, glucagon-stimulated) values could be of potential value in future decision-making regarding tapering or omitting insulin therapy in selected cases. The presence of pancreatic autoantibodies, detectable in ~50% of patients, is not an absolute requirement for the diagnosis and treatment of checkpoint inhibitor-associated diabetes. The management is based mainly on clinical expertise with these novel drugs. Insulin is the default therapy for glucose control, together with supportive measures (i.e. hydration, correction of electrolytes) according to standard guidelines (5, 117). There is currently no effective way of preventing or limiting the onset of this side effect. The attempt of immunomodulation with high-dose corticosteroids (i.e. standard treatment of irAE) was unsuccessful in reversing autoimmune diabetes following immunotherapy in a patient described by Aleksova *et al*. (60). Although omitting checkpoint blockade has been found to prevent further β cell loss in a single patient (118), given the compelling indication of immunotherapy in advanced malignancies with few treatment options, this might not be practically possible. This should perhaps be considered in an adjuvant setting where the prognosis is better. Restarting treatment with ICI should be considered once adequate glucose control has been established (5, 117). Finally, we acknowledge the need for further prospective studies to reassess/reevaluate current policy and expand our knowledge of the pathophysiology of this unique entity of diabetes mellitus associated with checkpoint inhibitors. Further exploration of risk factors and biomarkers is required to better identify individuals at risk and ideally prevent the onset of this rare but often aggressive form of diabetes.

## Conclusion

Checkpoint blockade-induced diabetes mellitus is a rare but potentially lethal AE, as diabetic ketoacidosis is often the first presentation. Despite its rarity, health-care professionals should be aware and patients need to be educated. This is crucial since a growing number of patients are treated with checkpoint blockade. Apart from raising awareness, periodic measurement of blood glucose is a practical screening option, for the time being. Predisposing factors, such as HLA genotype, may explain why some individuals are at greater risk. Our current knowledge of biomarkers, for the stratification of patients that need close follow-up, remains insufficient.

## Supplementary Material

Supplemental Table 1. Search terms (PDF 16 KB)

## Declaration of interest

Jeroen M K de Filette: lecture honoraria from Bristol-Myers Squibb. Lore Decoster: received grants from Boehringer-Ingelheim, Roche, Bristol-Myers Squibb. Sandrine Aspeslagh: speaker’s fees from Bristol-Myers Squibb, Roche, Astra Zeneca, Merck and Novartis. Bart Neyns: Honoraria – Bristol-Myers Squibb; Merck Sharp & Dohme; Novartis; Roche, Consulting or Advisory Role – Bristol-Myers Squibb; Merck Sharp & Dohme; Novartis; Roche Speakers’ Bureau – Novartis Travel, Accommodations, Expenses – Amgen; Bristol-Myers Squibb; Merck Sharp & Dohme; Novartis; Roche. The other authors have nothing to disclose.

## Funding

This research did not receive any specific grant from funding agencies in the public, commercial or not-for-profit-sector.

## Author contribution statement

J M K d F, A V K and B V planned the concept of this review. J M K d F, T V and A V K carried out the literature search. J M K d F and A V K performed the manuscript selection, data extraction and analysis. B J V D A performed HLA genotyping. J M K d F, B J V D A and A V K categorized the HLA genotypes. J M K d F and A V K drafted the manuscript. B O R provided the figure explaining the pathophysiology. All authors critically reviewed, revised and contributed to the final article.
