# Supplementary material for: Immune checkpoint inhibitors and type 1 diabetes mellitus: a case report and systematic review
Source: Eur J Endocrinol. 2019 Jul 19;181(3):363–74. doi: 10.1530/EJE-19-0291 (PMC6709545; doi:10.1530/EJE-19-0291)
Supplement: Supplemental Table 1. Search terms (PDF 16 KB) [file supplementary_table_1.pdf]

Supplemental Table 1. Search terms

1. *PubMed search terms*

("ipilimumab"[Supplementary Concept] OR ipilimumab*[tw] OR "tremelimumab"[Supplementary Concept] OR ticilimumab*[tiab] OR tremelimumab*[tw] OR "pembrolizumab"[Supplementary Concept] OR pembrolizumab*[tw] OR "nivolumab"[Supplementary Concept] OR nivolumab*[tw] OR "atezolizumab"[Supplementary Concept] OR atezolizumab*[tw] OR "avelumab"[Supplementary Concept] OR avelumab*[tw] OR "durvalumab"[Supplementary Concept] OR durvalumab*[tw]) AND ("Diabetes Mellitus"[Mesh:NoExp] OR diabet*[tiab] OR "Diabetes Mellitus, Type 1"[Mesh] OR (("type1"[ti] OR "typeI"[ti] OR "type 1"[ti] OR "type I"[ti] OR type one*[ti]) AND diabet*[ti]) OR t-1-d-m*[ti] OR t-I-d-m*[ti] OR t-1-dm*[ti] OR t-I-dm*[ti] OR t1-dm*[ti] OR tI-dm*[ti] OR t1dm*[ti] OR tIdm*[ti] OR "t1 d"[ti] OR "tI d"[ti] OR "t1d"[ti] OR "tId"[ti] OR t-1-diabet*[tiab] OR t-I-diabet*[tiab] OR t1 diabet*[tiab] OR tI diabet*[tiab] OR type 1 diabet*[tiab] OR type I diabet*[tiab] OR type one diabet*[tiab] OR type1 diabet*[tiab] OR typeI-diabet*[tiab] OR diabetes-type1*[tiab] OR diabetes-typeI*[tiab] OR diabetes type 1*[tiab] OR "diabetes type I"[tiab] OR diabetes-type-one*[tiab] OR "diabetes mellitus 1"[tiab] OR "diabetes mellitus I"[tiab] OR diabetes mellitus type1*[tiab] OR "diabetes mellitus typeI"[tiab] OR diabetes mellitus type 1*[tiab] OR "diabetes mellitus type I"[tiab] OR diabetes mellitus type one*[tiab] OR dm-type1*[tiab] OR "dm-typeI"[tiab] OR dm type 1*[tiab] OR "dm type I"[tiab] OR dm-type-one*[tiab] OR iddm*[ti] OR ((insulin depend*[tiab] OR insulindepend*[tiab]) AND (diabet*[tiab] OR diabetes*[all fields] OR diabetic*[all fields] OR diabetol*[all fields])) OR ((insulin therap*[tiab] OR insulintherap*[tiab]) AND (diabet*[tiab] OR diabetes*[all fields] OR

diabetic*[all fields] OR diabetol*[all fields])) OR ((insulin treat*[tiab] OR insulintreat*[tiab]) AND (diabet*[tiab] OR diabetes*[all fields] OR diabetic*[all fields] OR diabetol*[all fields])) OR ((intensive insulin*[tiab] OR insulin intens*[tiab] OR insulinintens*[tiab]) AND (diabet*[tiab] OR diabetes*[all fields] OR diabetic*[all fields] OR diabetol*[all fields])) OR insulin replac*[tiab] OR (brittle*[tiab] AND diabet*[tiab]) OR (juvenil*[tiab] AND diabet*[tiab]) OR (onset*[ti] AND diabet*[ti]) OR onset diabet*[tiab] OR autoimmune diabet*[tiab] OR auto-immune diabet*[tiab]) AND english[lang]

2. *Web of Science search terms*

(TS=("ipilimumab*" OR "ticilimumab*" OR "tremelimumab*" OR "pembrolizumab*" OR "nivolumab*" OR "atezolizumab*" OR "avelumab*" OR "durvalumab*")) AND (TI=(("type1" OR "typeI" OR "type-1" OR "type-I" OR "type-one*") AND "diabet*") OR TI=("t-1-d-m*" OR "t-I-d-m*" OR "t-1-dm*" OR "t-I-dm*" OR "t1-dm*" OR "tI-dm*" OR "t1dm*" OR "tIdm*" OR "t1-d" OR "tI-d" OR "t1d" OR "tId") OR TS=("diabet*" OR "t-1-diabet*" OR "t-I-diabet*" OR "t1-diabet*" OR "tI-diabet*" OR "type-1-diabet*" OR "type-I-diabet*" OR "type-one-diabet*" OR "type1-diabet*" OR "typeI-diabet*" OR "diabetes-type1*" OR "diabetes-typeI*" OR "diabetes-type-1*" OR "diabetes-type-I" OR "diabetes-type-one*" OR "diabetes-mellitus-1" OR "diabetes mellitus I" OR "diabetes-mellitus-type1*" OR "diabetes-mellitus-typeI" OR "diabetes-mellitus-type-1*" OR "diabetes-mellitus-type-I" OR "diabetes-mellitus-type-one*" OR "dm-type1*" OR "dm-typeI" OR "dm-type-1*" OR "dm-type I" OR "dm-type-one*") OR TI="iddm*" OR TS=(("insulin-depend*" OR "insulindepend*") AND ("diabet*" OR "diabetes*" OR "diabetic*" OR "diabetol*")) OR TS=(("insulin-therap*" OR "insulintherap*") AND ("diabet*"

OR "diabetes*" OR "diabetic*" OR "diabetol*")) OR TS=(("insulin-treat*" OR "insulintreat*") AND ("diabet*" OR "diabetes*" OR "diabetic*" OR "diabetol*")) OR TS=(("intensive-insulin*" OR "insulin-intens*" OR "insulinintens*") AND ("diabet*" OR "diabetes*" OR "diabetic*" OR "diabetol*")) OR TS="insulin-replac*" OR TS=("brittle*" AND "diabet*") OR TS=("juvenil*" AND "diabet*") OR TI=("onset*" AND "diabet*") OR TS=("onset-diabet*" OR "autoimmune diabet*" OR "auto-immune diabet*")) AND LA=english

3. *Cochrane search terms*

((ipilimumab* OR ticilimumab* OR tremelimumab* OR pembrolizumab* OR nivolumab* OR atezolizumab* OR avelumab* OR durvalumab*):ti,ab,kw) AND (((type1 OR typeI OR (type NEXT 1) OR (type NEXT I) OR (type NEXT one*)) AND diabet*):ti OR ((t NEXT 1 NEXT d NEXT m*) OR (t NEXT I NEXT d NEXT m*) OR (t NEXT 1 NEXT dm*) OR (t NEXT I NEXT dm*) OR (t1 NEXT dm*) OR (tI NEXT dm*) OR t1dm* OR tIdm* OR (t1 NEXT d) OR (tI NEXT d) OR t1d OR tId):ti OR (diabet* OR (t NEXT 1 NEXT diabet*) OR (t NEXT I NEXT diabet*) OR (t1 NEXT diabet*) OR (tI NEXT diabet*) OR (type NEXT 1 NEXT diabet*) OR (type NEXT I NEXT diabet*) OR (type NEXT one NEXT diabet*) OR (type1 NEXT diabet*) OR (typeI NEXT diabet*) OR (diabetes NEXT type1*) OR (diabetes NEXT typeI*) OR (diabetes NEXT type NEXT 1*) OR (diabetes NEXT type NEXT I) OR (diabetes NEXT type NEXT one*) OR (diabetes NEXT mellitus NEXT 1) OR (diabetes NEXT mellitus NEXT I) OR (diabetes NEXT mellitus NEXT type1*) OR (diabetes NEXT mellitus NEXT typeI) OR (diabetes NEXT mellitus NEXT type NEXT 1*) OR (diabetes NEXT mellitus NEXT type NEXT I) OR (diabetes NEXT mellitus NEXT type NEXT one*) OR (dm NEXT type1*) OR

(dm NEXT typeI) OR (dm NEXT type NEXT 1*) OR (dm NEXT type NEXT I) OR (dm NEXT type NEXT one*)):ti,ab,kw OR iddm*:ti OR (((insulin NEXT depend*) OR insulindepend*) AND diabet*):ti,ab,kw OR (((insulin NEXT therap*) OR insulintherap*) AND diabet*):ti,ab,kw OR (((insulin NEXT treat*) OR insulintreat*) AND diabet*):ti,ab,kw OR (((intensive NEXT insulin*) OR (insulin NEXT intens*) OR insulinintens*) AND diabet*):ti,ab,kw OR (insulin NEXT replac*):ti,ab,kw OR (brittle* AND diabet*):ti,ab,kw OR (juvenil* AND diabet*):ti,ab,kw OR (onset* AND diabet*):ti OR ((onset NEXT diabet*) OR (autoimmune NEXT diabet*) OR (auto NEXT immune NEXT diabet*)):ti,ab,kw)
